# Hemangioma in a pulmonary hilar lymph node: Case report

**DOI:** 10.1186/1477-7819-9-8

**Published:** 2011-01-26

**Authors:** Taichiro Goto, Kumi Akanabe, Arafumi Maeshima, Ryoichi Kato

**Affiliations:** 1 Department of General Thoracic Surgery, National Hospital Organization Tokyo Medical Center, Tokyo, Japan; 2 Department of Pathology, National Hospital Organization Tokyo Medical Center, Tokyo, Japan

## Abstract

**Background:**

Different types of vascular proliferation may occur in lymph nodes, but hemangiomas in lymph nodes are extremely rare.

**Case Presentation:**

A 73-year-old man was found to have a 15-mm nodular shadow in the left lung on computed tomography, and bronchoscopic brush cytology yielded a diagnosis of squamous cell carcinoma. Chest computed tomography showed no evidence of hilar or mediastinal lymphadenopathy. Left lower lobectomy with hilar and mediastinal lymph node dissection was performed. Postoperative histopathological examination revealed squamous cell carcinoma and no lymph node metastasis. On the other hand, a lobar bronchial lymph node presented a small lesion showing the dense proliferation of capillary blood vessels with elastic change. Immunohistochemically, the lesion was positive for factor VIII and CD34, leading to a diagnosis of primary hemangioma of the lymph node.

**Conclusion:**

To our knowledge, this is the first case reported in the literature of hemangioma in a pulmonary hilar lymph node. Intranodal hemangioma needs to be differentiated from malignant vascular tumors.

## Background

Although lymph nodes frequently display extensive vascularity in association with many different infections and other disease processes, the identification of a primary vascular tumor in a lymph node is a rare occurrence [1]. To date, only 20 cases have been published as intranodal hemangioma [1-6]. As rightfully stated by Almagro et al., these should be reported because of their unclear natural significance [3]. We present a case of a primary intranodal capillary/cavernous hemangioma and the first case documented to occur within a pulmonary hilar lymph node.

## Case Presentation

**Figure 1:**
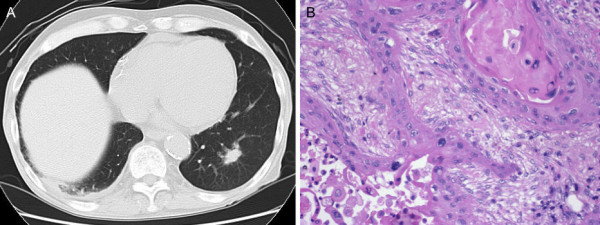
**Radiological and pathological findings of lung cancer**. A, Chest CT revealed a 15-mm nodular shadow in the left lower lobe. B, Histopathologically, the lesion was diagnosed as squamous cell carcinoma with stratification and keratinization.

**Figure 2:**
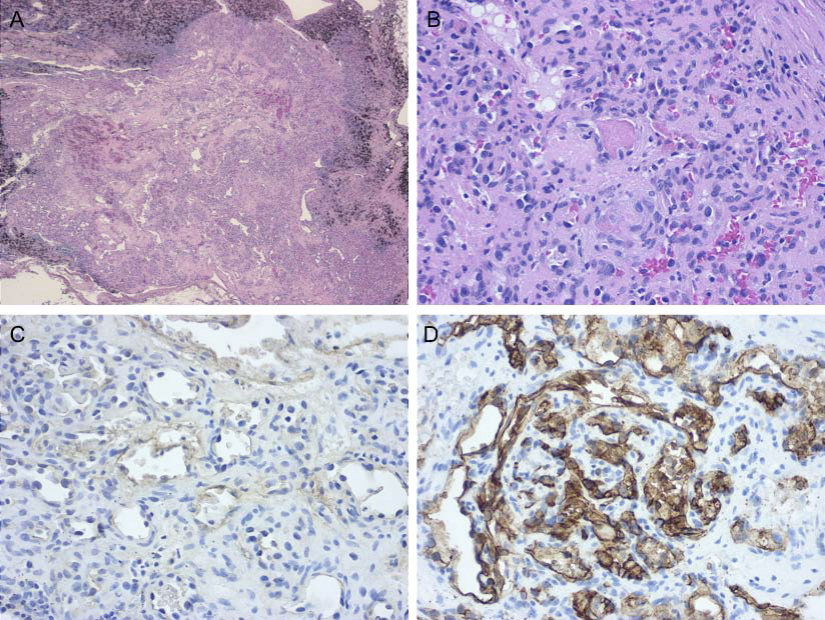
**Histological findings of a pulmonary hilar lymph node**. A, The lymph node exhibits residual nodal tissue and replacement by a vascular tumor (hematoxylin-eosin staining). B, The lesion is composed of well-developed capillaries. Organizing thrombi were identifiable (hematoxylin-eosin staining). C-D, Endothelial cells were positive for factor VIII and CD34 (C, Factor VIII immunostaining; D, CD34 immunostaining).

The patient was a 73-year-old man who was on dialysis for chronic renal failure in our hospital. He experienced pain in the left buccal mucosa, and visited the Department of Oral Surgery of our hospital. Biopsy revealed squamous cell carcinoma. Under a diagnosis of buccal mucosal cancer, he underwent arterial injection chemotherapy and radiation therapy in the Department of Oral Surgery. In addition, he was found to have a 15-mm tumor with an irregular margin in the left S9 on chest computed tomography (CT), and was referred to our department (Figure 1A). Bronchoscopic brush cytology led to a diagnosis of squamous cell carcinoma. The lung tumor showed histological features similar to those of the buccal mucosal cancer, but it was clinically diagnosed as a primary cancer because of its morphology on CT and because the buccal mucosal cancer was an early cancer. Chest CT showed no evidence of hilar or mediastinal lymphadenopathy. Under a diagnosis of lung cancer, left lower lobectomy with hilar and mediastinal lymph node dissection was performed. Postoperative histopathological examination revealed squamous cell carcinoma with stratification and keratinization and no lymph node metastasis. Thus, the tumor was diagnosed as pT1aN0M0, stage IA (Figure 1B). On the other hand, a lobar bronchial lymph node presented a lesion showing the dense proliferation of well-formed capillaries (Figure 2A, B). The stroma of the lesion showed small areas of fibrosis (Figure 2A). The lesion was well circumscribed and its borders sharply demarcated from the nodal lymphoid tissue (Figure 2A). There were organizing thrombi in many of the component capillaries (Figure 2B). There was no nuclear atypia and less mitotic activity. Immunohistochemically, the lesion was positive for factor VIII, α-smooth muscle actin and CD34, and negative for D2-40, cytokeratin AE 1/3, and CD68, leading to a diagnosis of primary hemangioma of the lymph node (Figure 2C, D).

His postoperative course was uneventful. At present, 20 months after surgery, he remains free of disease.

## Discussion

Although hemangiomas occur predominantly in the skin of the trunk and extremities, these lesions are actually ubiquitous in the human body. They have been observed in virtually all internal organs and are considered to be the most common benign neoplasms encountered in the liver. As pointed out by Almagro et al., there is no reason why they cannot occur as primary tumors of lymph nodes [3]. However, in fact, lymph node hemangiomas are rare, and only 20 cases of intranodal hemangioma, including ours, have been reported in the literature [1-6]. The lymph node sites in these 20 cases included the axillary, common iliac, supraclavicular, submental, inguinal, obturator, mesocolonic, submandibular, oral, cervical, and pulmonary hilar nodes [1-6]. Intranodal hemangiomas present as an asymptomatic, solitary palpable lymph node, or they may be an incidental finding [4]. A few examples of nodal hemangioma have been reported, which have occurred within nodes of sites draining malignant tumors. In addition to our case, radical mastectomy for breast carcinoma, salpingo-oophorectomy for ovarian cancer, and radical hysterectomy for endometrial carcinoma have yielded examples of capillary/cavernous hemangiomas in regional lymph nodes [4-6]. Intranodal hemangiomas have also been found in association with other vascular lesions, such as intestinal angiodysplasia and oral hemangiopericytoma [3]. This relationship suggests an angiogenic influence [6].

The case reported here is the first example of a hemangioma occurring in a pulmonary hilar lymph node. The diagnosis of hemangioma was made for the following reasons: The lesion had borders distinct from the surrounding nodal tissue. It was composed of well-formed capillaries identical to those of hemangiomas. The presence of organizing thrombi was observed in many component capillaries. The stroma of the lesion showed small areas of fibrosis. Malignant neoplastic features were not found in the lesion. Histologically, intranodal hemangiomas have been subclassified into 4 types: capillary/cavernous, cellular, lobular, and epithelioid [6,7], and in our case, the lymph node contained a capillary/cavernous hemangioma, defined as a benign vascular proliferation composed of small, capillary-sized blood vessels. A causative relationship between intranodal hemangioma and lung cancer has not been established, but it is generally considered that, in nodal angiomatosis, other associated lesions are present, most notably carcinoma [8]. Similarly, the development of hemangiomas in draining lymph nodes and the nature of their association with cancers are unknown, but we believe that the lesions are distinct from hemangiomas of an isolated nature. These reactive lesions may provide a clue to an underlying disease process, such as cancer, in the vicinity. Indeed, this syndromatic nature may be confirmed in the future with the accumulation of additional cases.

Hemangiomas are benign and, therefore, must be distinguished from malignant vascular tumors that involve lymph nodes, especially Kaposi's sarcoma. With the recent increase in acquired immunodeficiency syndrome and the fact that this tumor can present initially in lymph nodes without evident cutaneous involvement [9], this distinction assumes an ever increasing importance. In general, the bland appearance of the vascular structures in nodal hemangioma together with the absence of increased cellularity, anaplasia, a high mitotic index, and extravasation of erythrocytes are the features that distinguish this lesion from Kaposi's sarcoma [6,7,10].

## Abbreviations

CT: computed tomography

## Consent

Written informed consent was obtained from the patient for the publication of this case presentation and accompanying images. A copy of the written consent is available for review by the Editor-in-Chief of this journal.

## Competing interests

The authors declare that they have no competing interests.

## Authors' contributions

TG wrote the manuscript. TG, KA, and RK performed surgery. AM carried out the pathological examination. RK was involved in the final editing. All authors approved the final manuscript.
